# Hydroxychloroquine Induces Apoptosis in Cholangiocarcinoma *via* Reactive Oxygen Species Accumulation Induced by Autophagy Inhibition

**DOI:** 10.3389/fmolb.2021.720370

**Published:** 2021-09-10

**Authors:** Jiaqi Chen, Qiaoya Pan, Yang Bai, Xuepeng Chen, Yi Zhou

**Affiliations:** ^1^ Stomatology Hospital, School of Stomatology, Zhejiang University School of Medicine, Clinical Research Center for Oral Diseases of Zhejiang Province, Key Laboratory of Oral Biomedical Research of Zhejiang Province, Cancer Center of Zhejiang University, Hangzhou, China; ^2^ Department of Surgery, The Second Affiliated Hospital, Zhejiang University School of Medicine, Hangzhou, China

**Keywords:** HCQ, cholangiocarcinoma, ROS, autophagy, TCGA

## Abstract

**Purpose:** Despite considerable efforts to improve treatment modalities for cholangiocarcinoma, a common form of malignant tumor, its long-term survival rate remains poor. Hydroxychloroquine (HCQ) is a 4-aminoquinoline derivative antimalarial drug that has antimalarial and autophagy inhibition effects and exhibits comprehensive therapeutic effects on various cancers. In this study, we aimed to explore the anticancer potential and the underlying molecular mechanism of HCQ in cholangiocarcinoma treatment *in vitro* and *in vivo*.

**Methods:** Autophagy-related genes (ARGs) were obtained from the Human Autophagy Database and Molecular Signatures Database, and the expression profiles of ARGs were downloaded from the database of The Cancer Genome Atlas. Different expression gene sets were performed using R software. The Gene Ontology and KEGG enrichment analyses were performed to reveal significantly enriched signaling pathways and to identify differentially expressed genes in cholangiocarcinoma tissues. HuCCT-1 and CCLP-1 cells were exposed to different concentrations of HCQ. Cell proliferation was detected by Cell Counting Kit-8 (CCK-8), colony formation, and 5-ethynyl-2′-deoxyuridine (EdU) assays. Cell apoptosis and cycle arrest were detected by the Live/Dead cell assay and flow cytometry (FCM). The inhibition of autophagy was observed using fluorescence microscopy. The reactive oxygen species levels were assessed by fluorescence microscopy and flow cytometry. The protein levels were determined by western blot. A cholangiocarcinoma cell line xenograft model was used to evaluate the antitumor activity of HCQ *in vivo*.

**Results:** Compared with normal tissues, there were 141 ARGs with an aberrant expression in cholangiocarcinoma tissues which were mainly enriched in autophagy-related processes. Inhibition of autophagy by HCQ effectively suppressed cholangiocarcinoma *in vitro* and *in vivo*. HCQ inhibited cell proliferation and induced apoptosis and cycle arrest *in vitro* by increasing ROS accumulation, which was involved in autophagy inhibition. The ROS scavenger reduced l-glutathione distinctly weakened HCQ-induced cell apoptosis and viability inhibition in cholangiocarcinoma cells. In addition, HCQ inhibited growth of cholangiocarcinoma cell line xenograft tumors.

**Conclusion:** HCQ could inhibit cell proliferation and induce apoptosis in cholangiocarcinoma by triggering ROS accumulation *via* autophagy inhibition, which makes HCQ a potential antitumor drug candidate for cholangiocarcinoma treatment.

## Introduction

Cholangiocarcinoma is a malignant carcinoma that originates from the bile duct epithelium, which is characterized by the hidden onset and atypical early symptoms (Bekaii-Saab et al., 2021). Therefore, most patients are already in advanced stage when they are diagnosed for the first time, which leads to poor prognosis (O'Rourke et al., 2021). The main therapy for cholangiocarcinoma patients is surgical treatment (Rizvi et al., 2018). However, only a small percentage of patients can undergo radical surgery, and the postoperative local recurrence and distant metastasis rates remain very high (Blechacz, 2017). Given this, new therapeutic methods such as targeted therapy and immunotherapy are gradually being developed to improve patients’ prognosis (Saeed et al., 2019). However, it is unfortunate that the treatment effect of cholangiocarcinoma remains unsatisfactory. Therefore, it is imperative to identify new strategies for the treatment of patients with cholangiocarcinoma.

Autophagy is an important type of programmed cell death, which involves the breakdown and the recycling of aging proteins as well as the damaged organelles such as mitochondria within cells (Onorati et al., 2018; Amaravadi et al., 2019; Xia et al., 2021). The reactive oxygen species (ROS) are accumulated when the mitochondrial membrane is damaged. Therefore, they in turn induce autophagy so as to promote the progress of advanced cancer (Chude and Amaravadi, 2017; Shi et al., 2017). As crucial modulators for cellular homeostasis, both of them are associated with the survival of tumor cells (Vera-Ramirez et al., 2018). Thus, targeting the autophagy progression by a non-specific inhibitor hydroxychloroquine (HCQ) in cholangiocarcinoma may supply new approaches for cancer prevention (Piao et al., 2017; Xu et al., 2018).

In recent years, autophagy-related gene (ARG) expression signatures have been used to construct a prognosis risk model in many types of tumors (Hu et al., 2020; Zhang et al., 2020). To our knowledge, ARGs were closely related to tumor prognosis (Shi et al., 2020). Here in this study, we performed a biological information analysis on the transcriptome information of The Cancer Genome Atlas (TCGA)-CHOL cohort to screen out the differentially expressed ARGs. Meanwhile, we investigated the anticancer activities of HCQ in cholangiocarcinoma as well as the underlying molecular mechanisms of the process, which could support previous evidence on the role of autophagy in the development of cholangiocarcinoma and the potential therapeutic effect of other autophagy inhibitors (i.e., chloroquine, 3-methyladenine, wortmannin) (Yothaisong et al., 2013; Perez-Montoyo, 2020; Lendvai et al., 2021).

## Materials and Methods

### Acquisition of Gene Expression Information and Autophagy-Related Gene Sets

Information of gene expression was downloaded from the TCGA-CHOL cohort database. ARGs were obtained from the Human Autophagy Database (HADB). Finally, 44 mRNA expression profiles and 232 ARGs were obtained.

### Identification of Differential Expression of Autophagy-Related Gene Sets and Enrichment Analysis

The “edgeR” package of R 4.0.3 software was used to analyze the differential expression of autophagy-related gene sets (DE-ARGs) in 35 cholangiocarcinoma samples and 9 normal samples. Gene Ontology (GO) functional enrichment and Kyoto Encyclopedia of Genes and Genomes (KEGG) signaling pathways were analyzed using the “clusterprofiler,” “ggplot2,” “org.HS.eg.db,” and “enrichplot” package of R 4.0.3 software.

### Materials

The materials used in this study are hydroxychloroquine sulfate (cat. no. H141480, Aladdin), primary antibodies against caspase 3 (cat. no. ab184787, Abcam), PARP1 (cat. no. ab191217 Abcam), GAPDH (cat. no. ab181602, Abcam), cyclin E2 (cat. no. 4132, Cell Signaling Technology), p21 Waf1/Cip1 (12D1) (cat. no. 2947, Cell Signaling Technology), p53 (cat. no. ab26), Beclin-1 (cat. no. ab210498, Abcam), SQSTM1/p62 (cat. no. ab56416, Abcam), LC3B (cat. no. ab192890, Abcam), and LC3B (cat. no. AF4650, Affinity), horseradish peroxidase–conjugated Goat Anti-Rabbit IgG (H + L) (cat. no. SA00001-2, Proteintech), horseradish peroxidase–conjugated Goat Anti-Mouse IgG (H + L) (cat. no. SA00001-1, Proteintech), and Alexa Fluor–conjugated Goat Anti-Rabbit IgG (H + L) (cat. no. ab150077, Abcam). The above antibodies were used at the recommended concentrations according to the manufacturer’s instructions.

### Cell Culture

Two human cholangiocarcinoma cell lines HuCCT-1 and CCLP-1 purchased from the American Type Culture Collection (Manassas, VA, United States) were cultured in RPMI 1640 medium (cat. no. 01-100-1A, Biological Industries) supplemented with 10% FBS (cat. no. 04-001-1B, Biological Industries) and incubated at 37°C and 5% CO_2_.

### Cell Counting Kit-8 Assay

For concentration-dependent cell viability experiments, HuCCT-1 and CCLP-1 cells were seeded into 96-well plates (4 × 10^3^ cells/well) 18 h (37°C; 5% CO_2_) prior to treatment with various concentrations (0, 30, 60, 90, 120, 150, 180, 210, 240, and 270 μmol/L) of HCQ for an additional 24 h (37°C; 5% CO_2_). A total of 10 μL Cell Counting Kit-8 (CCK-8, cat. no. C0037, Beyotime) was subsequently added into each well for an additional 1.5 h (37°C; 5% CO_2_), and the absorption value was measured using a spectrophotometer. For time-dependent cell viability experiments, the cells were plated into 96-well plates 18 h (37°C; 5% CO_2_) prior to treatment with the IC50 and 2*IC50 concentrations of HCQ for 0, 6, 12, 24, 48, and 72 h (37°C; 5% CO_2_), and the optical density was measured at different time points in the same way. Curves were fitted and analyzed using GraphPad Prism 8 software (GraphPad Software, Inc.).

### Colony Formation Assay

HuCCT-1 and CCLP-1 cells were digested, counted, and seeded in a six-well plate (Guangzhou Jet Bio-Filtration Co., Ltd., 3,000–5,000 cells per well) 18 h prior to treatment with the IC50 and 2*IC50 concentrations of HCQ for 24 h. For the l-glutathione–reduced (GSH) group, the cells were preincubated with GSH (3 mmol/L) for 2 h and then treated with HCQ for 24 h. Then, the cells were cultured for another few days and stained with crystal violet. A clone was defined as more than 50 cells.

### EdU Viability Assay

According to the manufacturer’s instructions, the Cell Light EdU Apollo 567 *in vitro* kit (Guangzhou RiboBio Co., Ltd.) was used to evaluate the effect of HCQ. After treatment with IC50 and 2*IC50 concentrations of HCQ for 24 h, HuCCT-1 and CCLP-1 cells were EdU labeled and incubated for 2 h. Then, Apollo and Hoechst 33342 staining was performed. Fluorescence microscopy (Olympus Corporation, Japan) was used to photograph the cells.

### Cell Apoptosis and Cell Cycle Analysis

Apoptosis was determined by an Annexin V-FITC Apoptosis Detection Kit (cat. no. C1062L, Beyotime). HuCCT-1 and CCLP-1 cells were seeded at a density of 4 × 10^5^ cells per well in six-well plates. Following exposure to IC50 and 2*IC50 concentrations of HCQ for 24 h, the cells were incubated with Annexin V-FITC (fluorescein isothiocyanate isomer) and propidium iodine (PI) for 20 min at 37°C in the dark according to the manufacturer’s protocol. For the GSH group, the cells were preincubated with GSH (3 mmol/L) for 2 h and then treated with HCQ for 24 h. Cell cycle distribution was determined by a Cell Cycle and Apoptosis Analysis Kit (cat. no. C1052, Beyotime). Following exposure to the IC50 concentration of HCQ for 24 h, the cells were collected and fixed with 75% precooled ethanol. Upon completion of treatment, the cells were stained with PI for 20 min according to the instructions. Finally, apoptosis and cell cycle distribution were examined by flow cytometry (FCM, BD Biosciences, United States) and analyzed by Flowjo software 10.6.2 (FlowJo LLC) and ModFit LT software 5 (Verity Software House, Inc.).

### Live/Dead Cell Assay

Live/Dead cells were determined by a Calcein/PI Cell Viability/Cytotoxicity Assay Kit (cat. no. C2015M, Beyotime). Following exposure to IC50 and 2*IC50 concentrations of HCQ for 24 h, HuCCT-1 and CCLP-1 cells were digested and stained with Calcein-AM and PI for 30 min at room temperature. Pictures were taken using a fluorescence microscope (Olympus IX71).

### Reactive Oxygen Species Detection

The accumulation of ROS was detected by an ROS assay kit (cat. no. S0033S, Beyotime). The cells were exposed to HCQ for 24 h. For the GSH group, the cells were preincubated with GSH (3 mmol/L) for 2 h and then treated with HCQ for 24 h. Then, the cells were collected and incubated with 10 μmol/L 2,7-dichlorodihydrofluorescein diacetate (DCFH-DA) at 37°C for 30 min and washed three times. The level of ROS was determined by fluorescence microscopy and FCM.

### Immunofluorescence Microscopy

After treatment with the IC50 concentration of HCQ for 24 h, HuCCT-1 and CCLP-1 cells were subsequently incubated with rabbit polyclonal antibody against LC3B (1:400) overnight at 4°C. Following addition of fluorochrome-conjugated anti-rabbit IgG (1:200) and incubation at 37°C for 1 h, DAPI counterstaining was performed at room temperature for 30 min. Pictures were taken using an Olympus IX71 digital camera.

### Autophagy Flux Detection

The cells were seeded in a confocal dish at a density of 10,000 cells per dish. Following exposure to adenovirus expressing mCherry-GFP-LC3B fusion protein recognizing CD46 (AdPlus-mCherry-GFP-LC3B, cat. no. C3012, Beyotime) for 48 h, the cells were incubated with the IC50 concentration of HCQ for 24 h. Pictures were taken using ZEISS LSM 900 confocal microscopy.

### Western Blot Analysis

Western blot analysis was performed as reported previously (Wang et al., 2021). HuCCT-1 and CCLP-1 cells were digested and dissolved in Radio Immunoprecipitation Assay (RIPA) Lysis Buffer (cat. no. P0013B, Beyotime) containing 1% protease inhibitor cocktail (cat. no. P8340, Sigma-Aldrich) for 1 h. An enhanced BCA Protein Assay Kit (cat. no. P0010, Beyotime) was then used to detect the protein concentration. Equal amounts of protein were then separated on 10% SurePAGE Bis-Tris gels (cat. no. M00664, GenScript) for electrophoresis and transferred to polyvinylidene fluoride (PVDF) membranes. Membranes were then blocked using a QuickBlock Blocking Buffer (cat. no. P0252, Beyotime) at 37°C for 1 h and incubated with primary antibodies against GAPDH, caspase 3, PARP1, cyclin E2, p21, p53, Beclin-1, LC3B, and SQSTM1/P62 at 1:1,000 overnight. Membranes were then incubated with anti-rabbit secondary antibodies at 1:2,500 for 1 h at room temperature, and protein bands were finally detected by an Enhanced Chemiluminescence Kit (cat. no. G2014, Servicebio). Band intensity was quantified using ImageJ software (National Institutes of Health).

### *In Vivo* Tumor Xenograft Models

Ten BALB/c nude mice (5 weeks old, 18–20 g) were housed under specific-pathogen–free (SPF) conditions. Approximately 5 × 10^6^ CCLP-1 cells were injected into the right back of each mouse subcutaneously to establish the tumor model. Until the tumors reached about 100 mm (Rizvi et al., 2018), the mice were randomly assigned into two groups: 1) control group (n = 5, 0.9% physiological saline) and 2) HCQ group (n = 5, 100 mg/kg). These compounds were administered intraperitoneally once daily. The body weight and tumor size were recorded during the treated period. The mice were sacrificed 3 weeks after injection.

### Immunohistochemistry Staining

The immunohistochemistry of the xenograft tumor was conducted as previously described (Cao et al., 2020). 2 mm thick sections were stained with hematoxylin and eosin (HE) for the analysis of inflammation and necrosis. Tumor tissues were subjected to immunohistochemical staining of Ki-67 for the analysis of proliferation ability. Apoptotic cells were determined by TdT-mediated dUTP nick-end labeling (TUNEL) technique with a TUNEL Apoptosis Assay Kit (cat. no. C1091, Beyotime) following the manufacturer’s instructions.

### Statistical Analysis

Data and figures were processed using GraphPad Prism 8 software (GraphPad Software, Inc.). All values were expressed as mean ± standard deviation (SD) and assessed using Student’s t-test or one-way analysis of variance procedure. Except for the *in vivo* tumor xenograft experiment (five mice were allocated to each group, so as to ensure the sufficient group size for statistical analysis) which was carried out only once, all other experiments were carried out in triplicate with at least three independent experiments. *p* < 0.05 was considered to indicate a statistically significant difference.

## Results

### Identification and Enrichment Analysis of DE-ARGs

**FIGURE 1:**
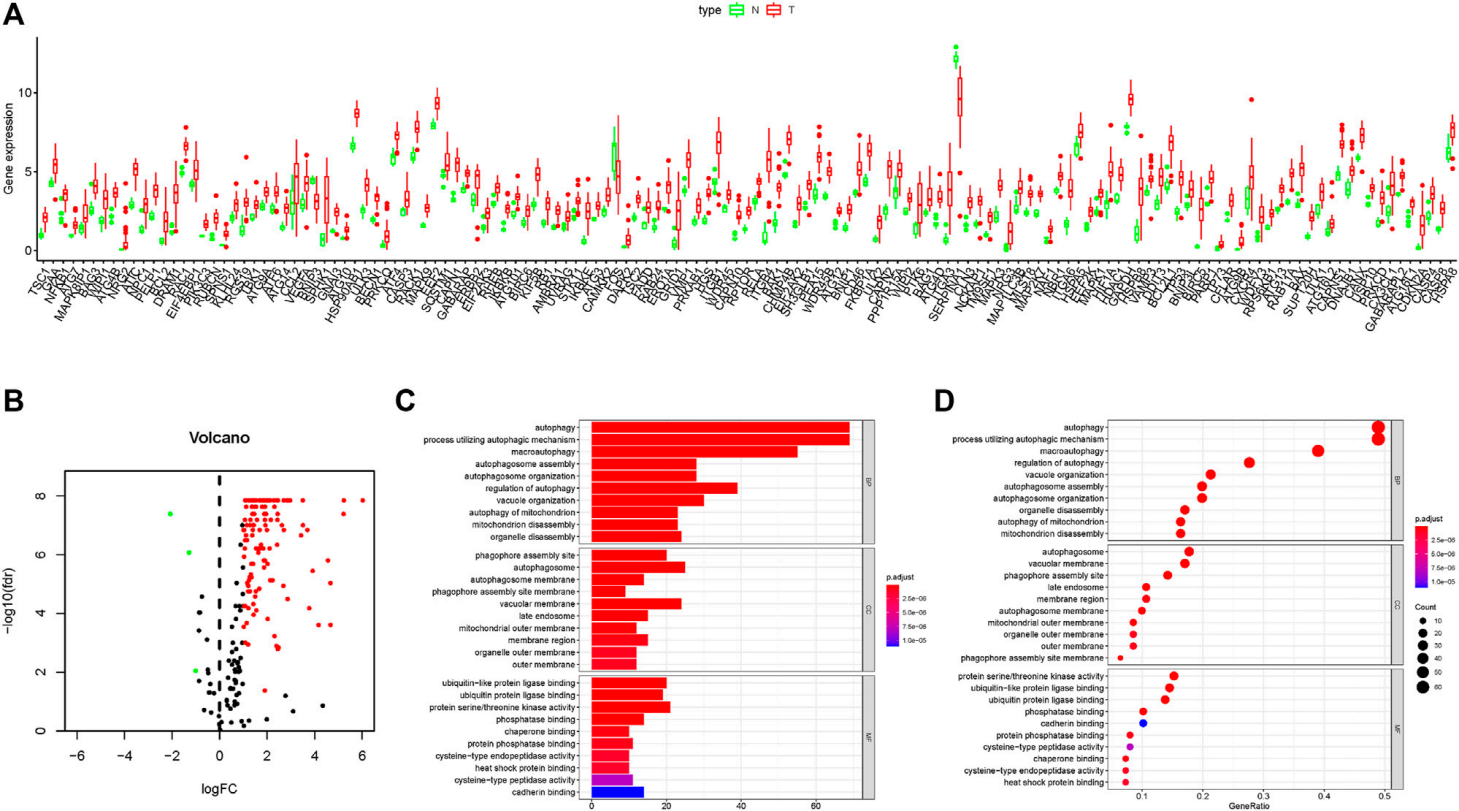
Results of DEG expression profiles and enrichment analysis. **(A–B)** Box plot and volcano plot of differentially expressed autophagy-related genes in cholangiocarcinoma tissues. **(C–D)** Bar plot and bubble chart of relationship between differentially expressed genes and functional pathways in GO analysis.

In this work, mRNA expression profiles with 35 cholangiocarcinoma samples and 9 normal samples were collected from the TCGA database, and 232 ARGs were obtained from the HADB. The heat map of DE-ARGs in cholangiocarcinoma is shown in Supplementary Figure 1A. Compared with the normal tissues, there were 141 DE-ARGs in cholangiocarcinoma tissues which contained 138 up-regulated ARGs and only 3 down-regulated ARGs (Figures 1A,B). GO functional enrichment analysis showed that DE-ARGs were mostly enriched in autophagy and phagophore assembly sites to perform the functions of biological processes and cell components, respectively (Figures 1C,D). Moreover, KEGG signaling pathway analysis showed that DE-ARGs mostly enriched in some programmed cell death signaling pathways such as autophagy and apoptosis (Supplementary Figures 1B,C). These results suggested that autophagy might play a crucial role in the development of cholangiocarcinoma, and the inhibition of it might have therapeutic effect on cholangiocarcinoma.

### Inhibition of Autophagy Reduced the Activity of Cholangiocarcinoma Cells

**FIGURE 2:**
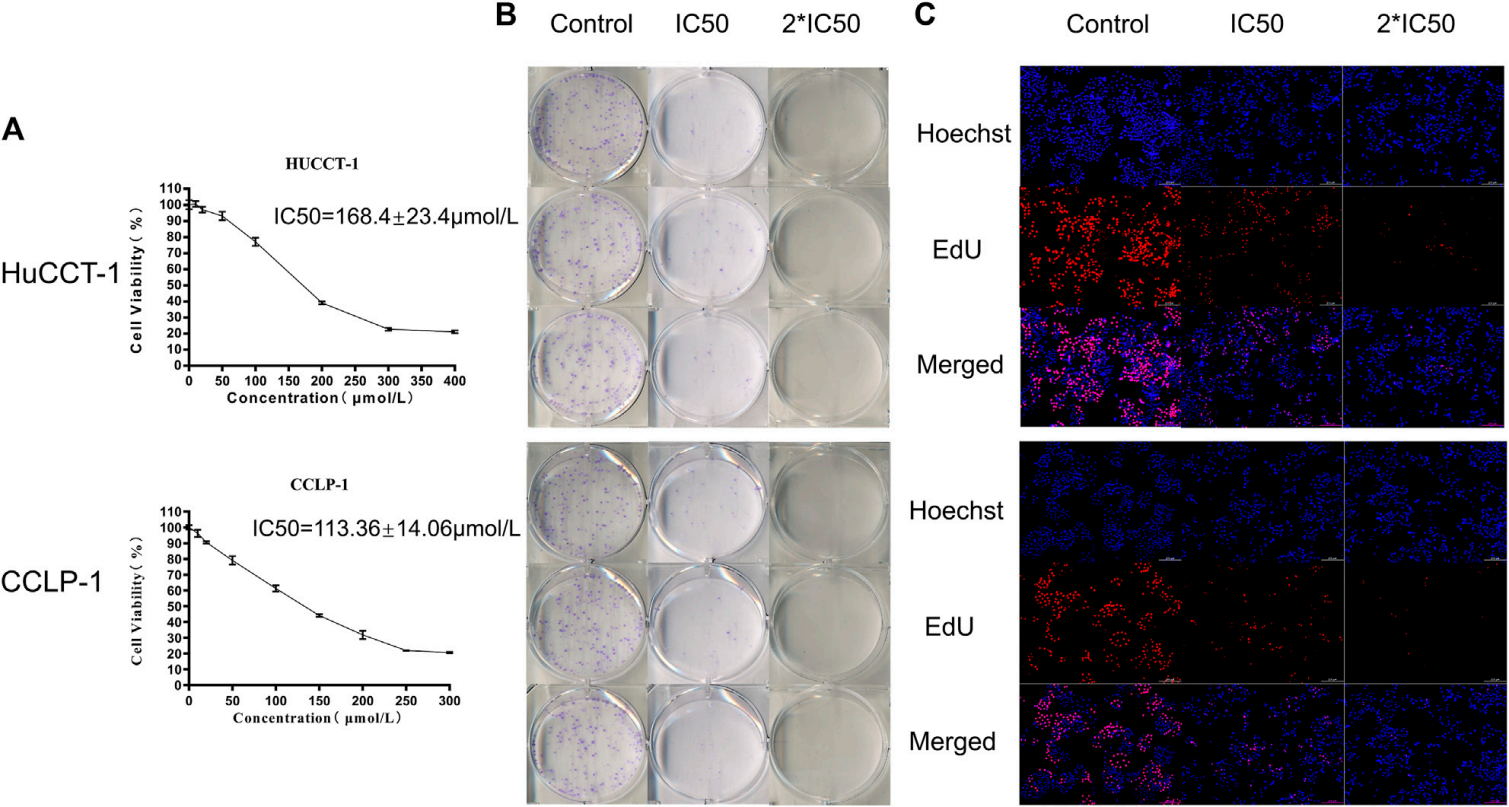
HCQ inhibits cell viability in HuCCT-1 and CCLP-1 cells. **(A)** HCQ inhibited human cholangiocarcinoma cell viability. HuCCT-1 and CCLP-1 cells were treated with HCQ in different concentrations for 24 h, and the cell viability was measured with the CCK-8 assay (n = 3). The IC50 concentration was calculated according to its proliferation inhibition rate. **(B–C)** Colony formation assay and EdU assay results of HuCCT-1 and CCLP-1 cells treated with IC50 and 2*IC50 concentrations of HCQ.

The above results confirmed that the autophagy process was active in cholangiocarcinoma tissues, so we hypothesized that inhibition of autophagy could have a therapeutic effect on tumors. The classic autophagy inhibitor HCQ was selected to treat two cholangiocarcinoma cell lines, HuCCT-1 and CCLP-1. As shown in Figure 2A, the cell viability of HuCCT-1 and CCLP-1 cells was significantly inhibited by HCQ, and the IC50 of the two cell lines was 168.4 ± 23.4 μmol/L and 113.36 ± 14.06 μmol/L, respectively. Moreover, cholangiocarcinoma cells were inhibited by HCQ in a time-dependent manner (Supplementary Figures 2A,B). The colony formation assay and EdU assay also indicated that the colony formation ability (for the IC50 group, *p* < 0.01 in HuCCT-1 and *p* < 0.05 in CCLP-1) and the proliferation ability (*p* < 0.01) of cholangiocarcinoma cells were significantly inhibited in HCQ-treated cells (Figures 2B,C, Supplementary Figures 2C,D).

### HCQ Induced Apoptosis in Cholangiocarcinoma Cells

**FIGURE 3:**
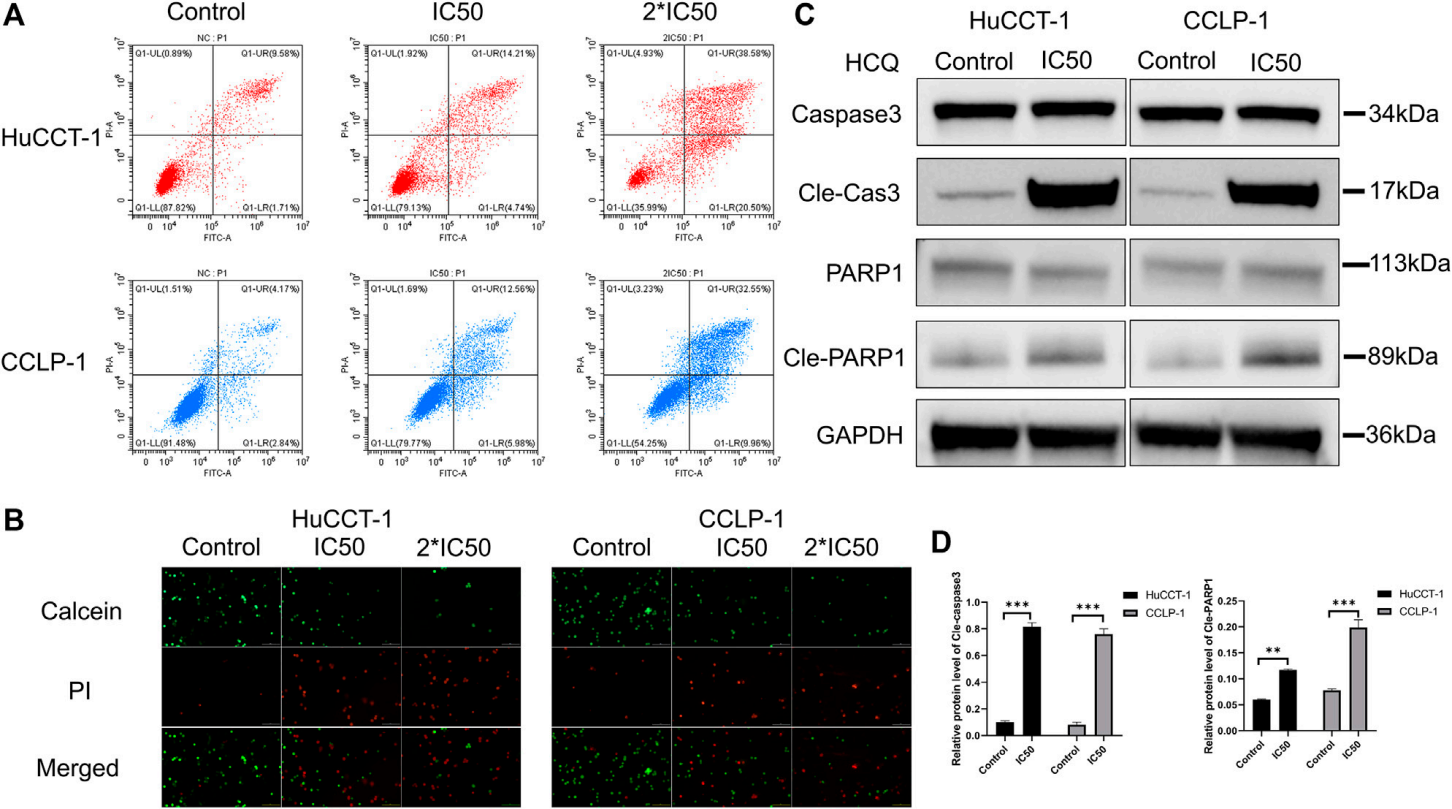
HCQ induces apoptosis in cholangiocarcinoma cells. **(A–B)** HuCCT-1 and CCLP-1 cells were treated with HCQ (IC50 and 2*IC50 concentrations) for 24 h, and apoptotic cells were analyzed by flow cytometry and Live/Dead cell assay. **(C)** Cells were treated with the IC50 concentration of HCQ for 24 h, and the apoptosis-related proteins were analyzed by western blotting. **(D)** The histograms indicate the relative protein level of cleaved caspase 3 and cleaved PARP1. All values are expressed as mean ± SD. **p < 0.01, ***p < 0.001, significant difference compared with the control group.

Cell death can significantly affect cell viability. After the treatment with IC50 and 2*IC50 concentrations of HCQ for 24 h, FCM with FITC/PI double staining was used to confirm the role of HCQ in inducing apoptosis in cholangiocarcinoma cells. Compared with the control group, apoptosis in the HuCCT-1 and CCLP-1 cells (for the IC50 group, *p* < 0.001 in HuCCT-1 and *p* < 0.01 in CCLP-1) increased in a dose-dependent manner after the 24 h HCQ treatment (Figure 3A, Supplementary Figures 3A,B). Consistent with the FCM results, the Live/Dead cell assay revealed a significant transition from the calcein signal to the PI signal (Figure 3B), indicating that HCQ-induced apoptosis increased with the increase of dose. To further determine whether apoptosis occurred instead of necrosis or other forms of cell death, we used western blotting (WB) to explore the expression of caspase 3 and PARP1, which were both downstream apoptosis-related proteins. As shown in Figures 3C,D, the expression of cleaved caspase 3 (*p* < 0.001) and cleaved PARP1 (*p* < 0.01 in HuCCT-1 and *p* < 0.001 in CCLP-1) increased significantly, while the expression of caspase 3 and PARP1 had no significant change. In brief, these data suggested that HCQ induced cholangiocarcinoma cell death by activating apoptosis pathways.

### HCQ Induced G1 Arrest in Cholangiocarcinoma Cells

**FIGURE 4:**
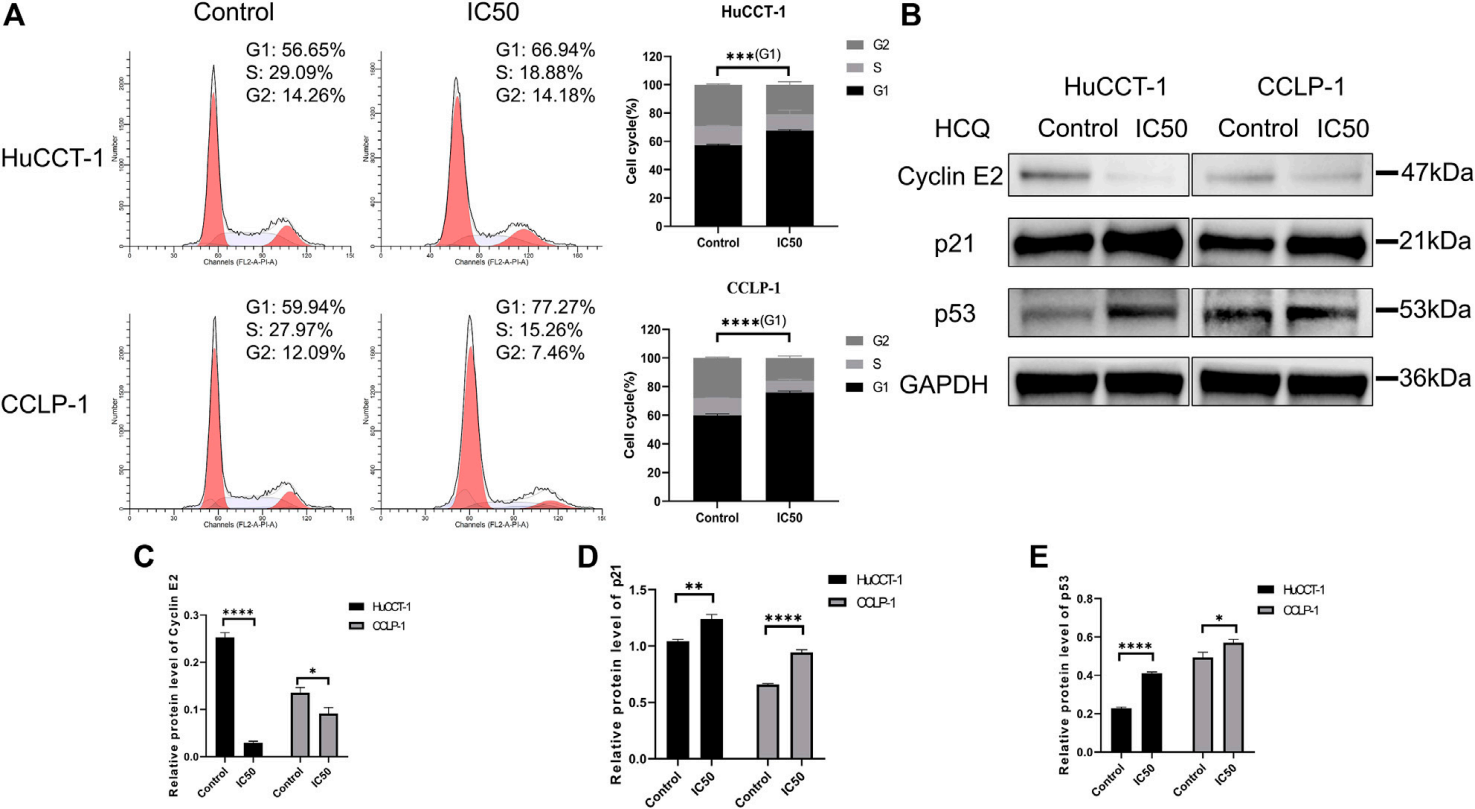
HCQ induces cell cycle arrest in the G1 phase of cholangiocarcinoma cells. **(A)** FCM analysis was used to detect cell cycle distribution after treatment with the IC50 concentration of HCQ, the histograms show the percentage of HuCCT-1 and CCLP-1 cells in the G1-S-G2 phase, and the comparison of G1 phase between two groups was analyzed using Student’s t-test. **(B)** The G1 checkpoint proteins were analyzed by WB after treatment with the IC50 concentration of HCQ for 24 h. **(C–E)** Quantification of WB results of cyclin E2, p21, and p53. Data are shown as mean ± SD. *p < 0.05, **p < 0.01, ***p < 0.001, ****p < 0.0001, significant difference compared with the control group.

To determine the underlying mechanism resulting in the suppression of cell proliferation and loss of cell viability, cell cycle distribution was assessed using FCM. As shown in Figure 4A, an increase in the percentage of cells in G1 phase was observed in HuCCT-1 and CCLP-1 cells after being treated with the IC50 concentration of HCQ for 24 h, from 56.65 to 66.94% (*p* < 0.001) and from 59.94 to 77.27% (*p* < 0.0001), respectively. Next, the expression of the G1 checkpoint protein was detected using WB. As presented in Figures 4B–E, p21 (*p* < 0.01 in HuCCT-1 and *p* < 0.0001 in CCLP-1) and p53 (*p* < 0.0001 in HuCCT-1 and *p* < 0.05 in CCLP-1) increased markedly, and a marked reduction was observed in cyclin E2 (*p* < 0.0001 in HuCCT-1 and *p* < 0.05 in CCLP-1), which indicated that treatment of HCQ with IC50 concentration might result in G1 arrest in cholangiocarcinoma cells.

### HCQ Induced ROS Accumulation by Inhibiting Autophagy

**FIGURE 5:**
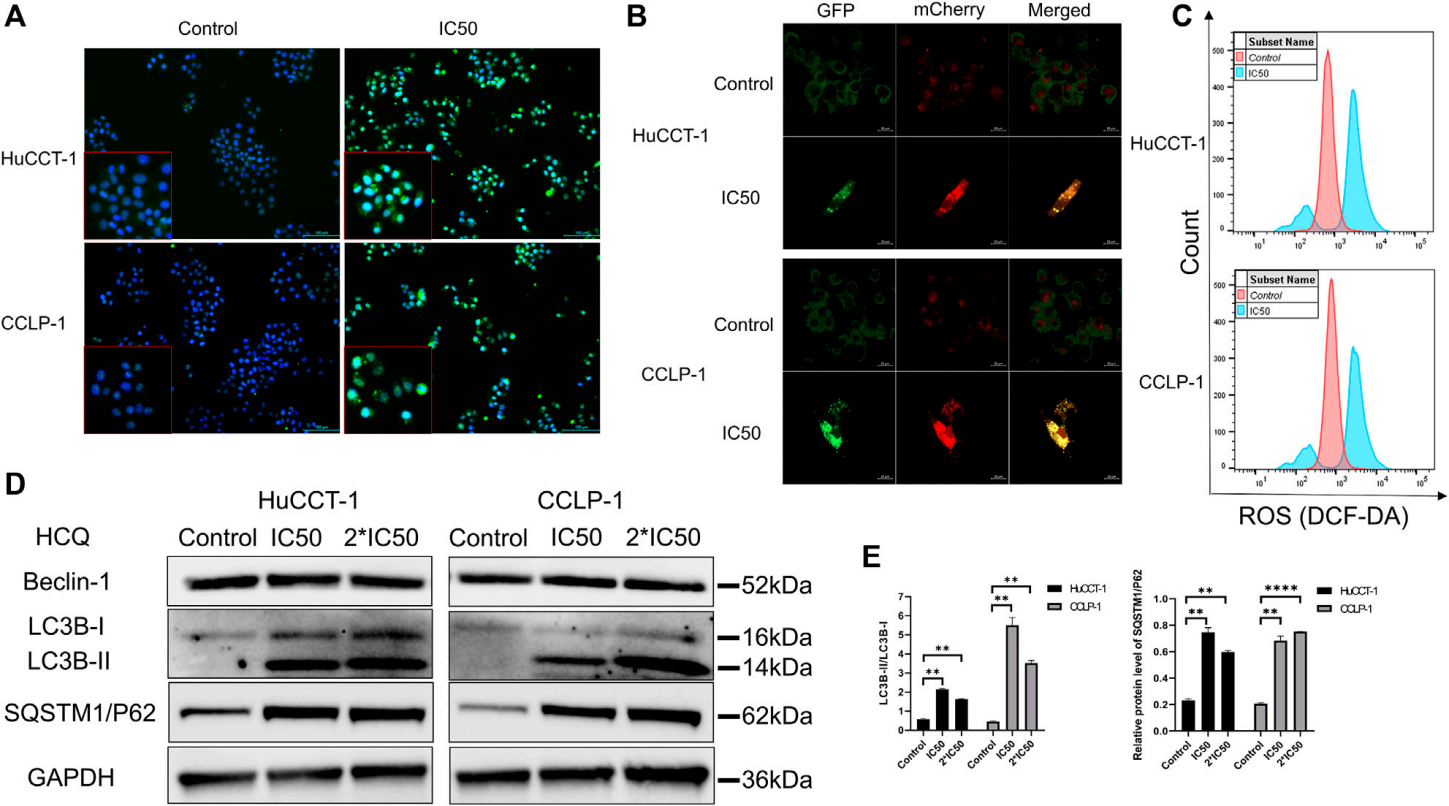
HCQ inhibits autophagy and causes ROS accumulation in cholangiocarcinoma cells. **(A)** Autophagy inhibition was observed by immunofluorescence microscopy (blue fluorescence indicates Hoechst and green fluorescence indicates LC3B puncta) after HCQ (IC50) treatment for 24 h. **(B)** Autophagy flux was inhibited in both cell lines after HCQ treatment. **(C)** ROS levels of HuCCT-1 and CCLP-1 cells treated with HCQ were determined by FCM. **(D)** Expression of Beclin-1, LC3B, and SQSTM1/P62 proteins was detected by western blotting in HuCCT-1 and CCLP-1 cells treated with IC50 and 2*IC50 concentrations of HCQ. **(E)** Quantification of LC3B and SQSTM1/P62 proteins. Data are expressed as mean ± SD. **p < 0.01, ****p < 0.0001, significant difference compared with the control group.

HCQ may inhibit autophagy to decrease the elimination of damaged organelles so as to increase the excessive production of ROS in cholangiocarcinoma cells. Autophagy inhibition and ROS content were analyzed using immunofluorescence (IF) microscopy and FCM. As shown in Figure 5A, microtubule-associated proteins light chain 3B (LC3B)-positive punctate dots (green fluorescence) increased markedly after HCQ (IC50) treatment in HuCCT-1 and CCLP-1 cells. After the treatment with the IC50 concentration of HCQ for 24 h, mCherry-GFP-LC3B was aggregated on the membrane of autophagosome in the form of yellow spots under a confocal microscope which means autophagy inhibition (Figure 5B). Correspondingly, the mean fluorescence intensity of 2′,7′-dichlorofluorescein (DCF) increased significantly after HCQ treatment in both cell types (Figure 5C, Supplementary Figures 4A,B, *p* < 0.0001 in HuCCT-1 and *p* < 0.01 in CCLP-1), suggesting that ROS had accumulated. When autophagy was inhibited, the degradation of LC3-II and sequestosome 1/P62(SQSTM1/P62) was reduced, while the process of LC3 conversion from LC3-I to LC3-II and SQSTM1/P62 synthesis continued. Therefore, autophagy was assessed using the content of SQSTM1/P62 and the LC3-II/LC3-I ratio by WB analysis. In Figures 5D,E, following HCQ (IC50 and 2*IC50) treatment, LC3B-II (*p* < 0.01) and SQSTM1/P62 (*p* < 0.01) increased markedly in both cell types, while Beclin-1 did not reveal any significant change.

### HCQ-Mediated Apoptosis of Cholangiocarcinoma Cells Occurred in an ROS-Dependent Manner

**FIGURE 6:**
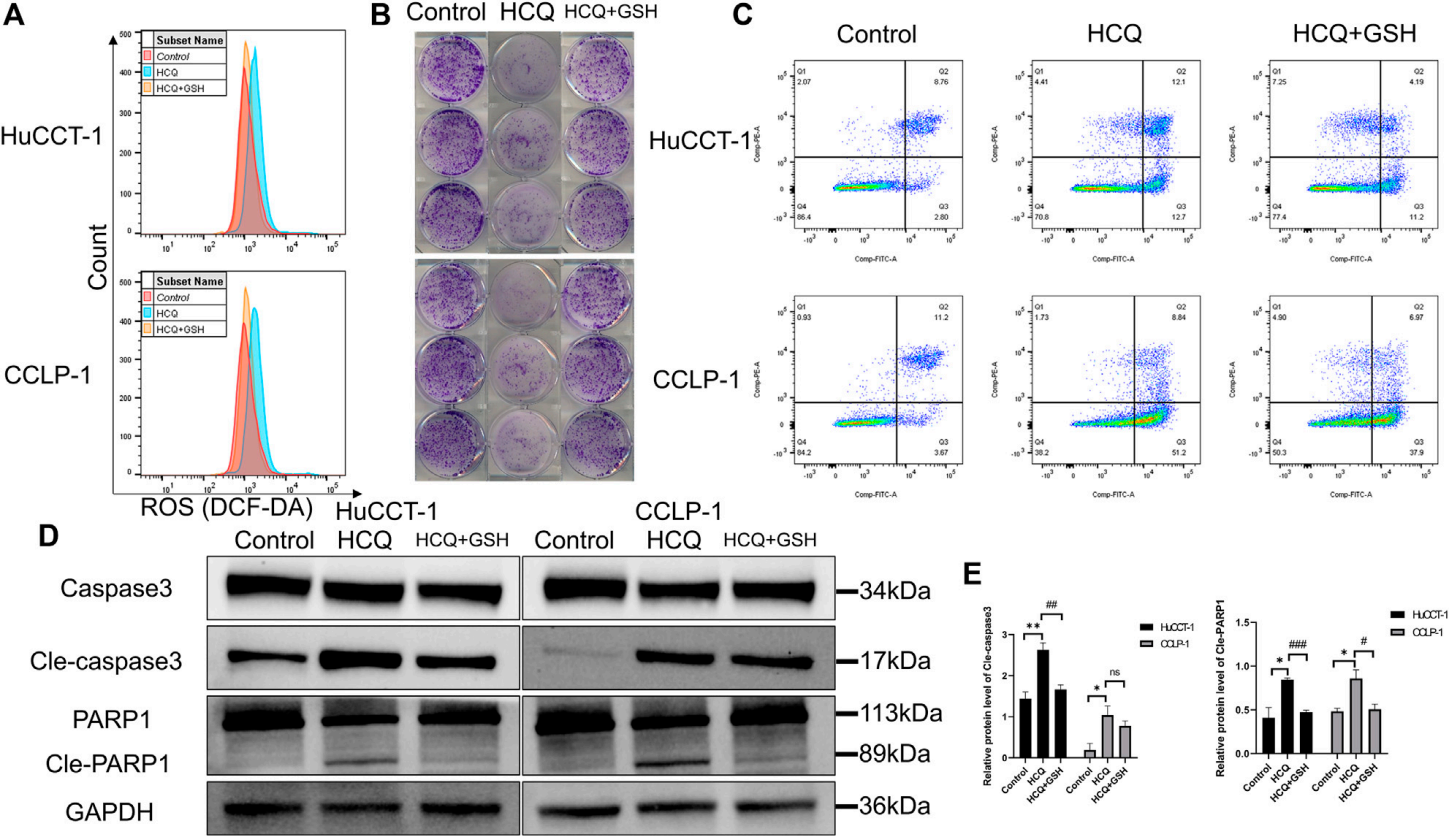
HCQ induces apoptosis in cholangiocarcinoma cells in an ROS-dependent manner. Cells were preincubated with GSH (3 mmol/L) for 2 h and then treated with HCQ for 24 h. **(A)** ROS levels in HuCCT-1 and CCLP-1 cells treated with HCQ (IC50) and GSH were detected by flow cytometry. **(B)** The effect of HCQ (IC50) on the colony formation capacity of HuCCT-1 and CCLP-1 cells was reversed by GSH treatment. **(C)** The effect of HCQ (IC50) on apoptosis of HuCCT-1 and CCLP-1 cells was reversed by GSH treatment. **(D)** After treatment with GSH, cleaved caspase 3 and cleaved PARP1 protein levels were decreased in HuCCT-1 and CCLP-1 cells. **(E)** Quantification of WB results of cleaved caspase 3 and cleaved PARP1. The results are expressed as mean ± SD. *p < 0.05, **p < 0.01, significant difference compared with the control group; ^#^ *p* < 0.05, ^##^ *p* < 0.01, ^###^ *p* < 0.001, significant difference compared with the HCQ group.

HCQ can induce apoptosis of cholangiocarcinoma and ROS accumulation. Therefore, we hypothesized that HCQ disrupted cholangiocarcinoma survival by inducing alterations in ROS levels. Subsequently, we examined the effects of l-glutathione–reduced (GSH) group, which scavenged ROS, on colony formation and apoptosis. As shown in Figure 6A and Supplementary Figure 5A, the increase of ROS levels in HuCCT-1 and CCLP-1 cells was reversed by GSH treatment (for the HCQ + GSH group, *p* < 0.01 in HuCCT-1 and *p* < 0.001 in CCLP-1). Colony formation and apoptosis analysis had produced similar results. The effect of HCQ on inhibition of cholangiocarcinoma cells was reversed by GSH treatment (Figures 6B,C, Supplementary Figure 5B). At the protein level, the increase in cleaved caspase 3 (for the HCQ + GSH group, *p* < 0.01 in HuCCT-1 and no significant change in CCLP-1) and cleaved PARP1 (for the HCQ + GSH group, *p* < 0.001 in HuCCT-1 and *p* < 0.05 in CCLP-1) in HuCCT-1 and CCLP-1 cells was reversed by HCQ treatment (Figures 6D,E). Collectively, our results demonstrated that GSH decreased ROS levels, which in turn reversed survival of cholangiocarcinoma cells.

### HCQ Suppressed *In Vivo* Growth of Tumors

**FIGURE 7:**
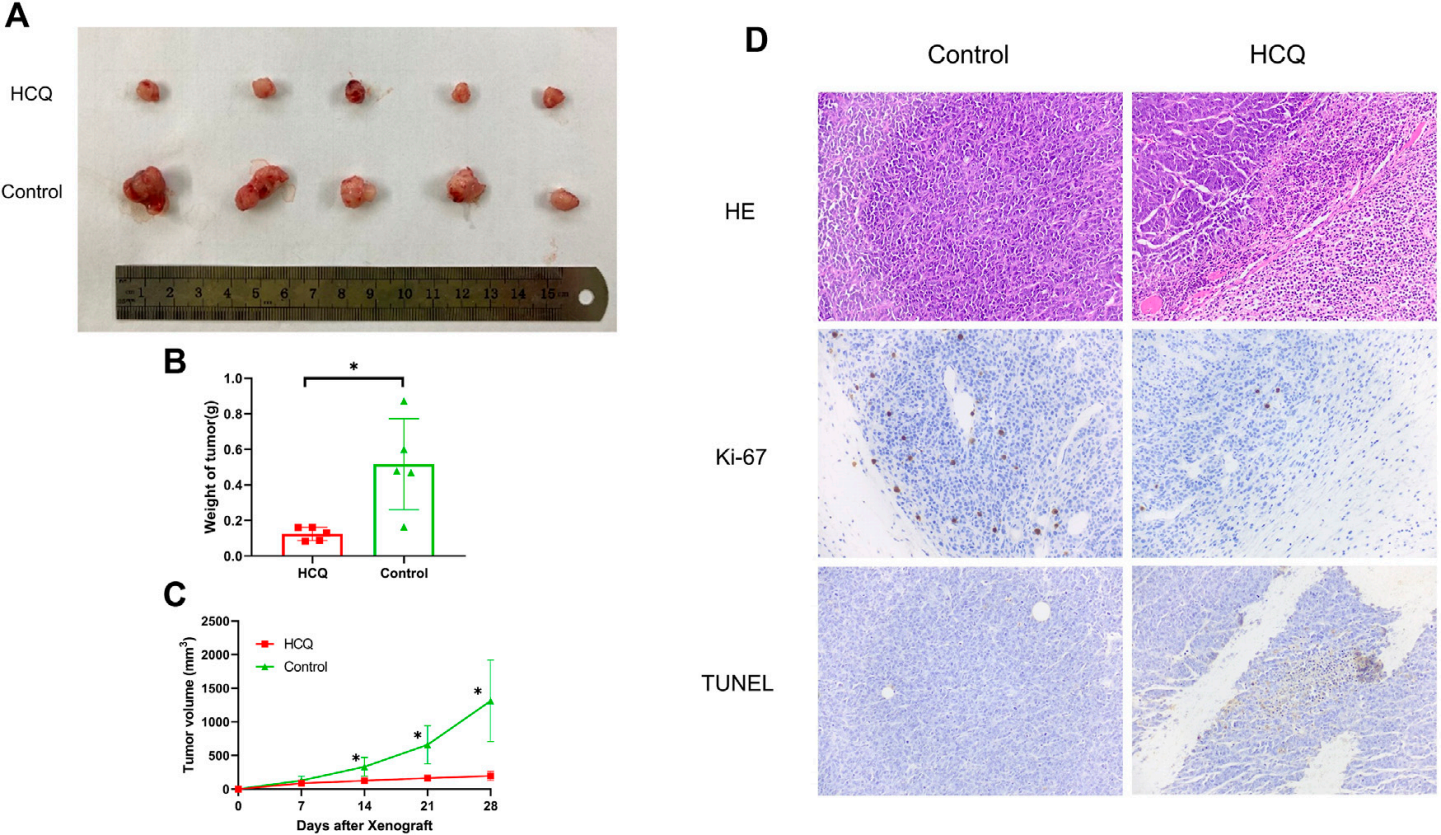
HCQ suppresses the tumor growth in the cholangiocarcinoma xenograft model. **(A)** Antitumor activity of HCQ (100 mg/kg) in nude mice, representative xenografts from mice after subcutaneous injection of CCLP-1 cells and treatment of HCQ. **(B)** Tumor weights of each group measured at the end of the treatment. Data are shown as mean ± SD; *p < 0.05 means significant difference compared with the control group. **(C)** Tumor volumes of each group measured in indicated days of treatment. All values are expressed as mean ± SD; *p < 0.05 means significant difference compared with the control group at the same time point. **(D)** HE, Ki-67, and TUNEL immunohistochemistry assays of tumor tissue. For the IHC results of Ki-67 and TUNEL, yellow stains mean proliferative cells and apoptotic cells, respectively.

To investigate the effect of HCQ on tumor growth *in vivo*, mice bearing cholangiocarcinoma subcutaneous xenografts were used. As shown in Figures 7A,B, we found that tumor weight was significantly reduced following HCQ treatment for 3 weeks. From the seventh day after HCQ administration, the growth rate of the tumor was significantly reduced compared to that observed in the control group, while there was no significant difference in body weight of mice between two groups (Figure 7C, Supplementary Figure 5C). IHC analysis revealed a dramatic reduction of Ki-67 staining and significantly increase of TUNEL staining in the tumor tissues of mice treated with HCQ (Figure 7D) which meant that the proliferation of cholangiocarcinoma was inhibited and apoptosis played a huge role in it. In summary, our results suggested that HCQ inhibited cholangiocarcinoma growth *in vivo*.

## Discussion

Nowadays, several prognostic factors are identified to determine cholangiocarcinoma prognosis, including the ceRNA regulatory network (Xia et al., 2020) and DNA methylation (Nakaoka et al., 2017). Meanwhile, high-throughput sequencing datasets (Jeon et al., 2018) or several effective biomarkers (Lang et al., 2021) are being used to explore potential molecular mechanisms in cholangiocarcinoma treatment. Due to its comprehensive genetic heterogeneity, the key drivers involved in the genesis and development of cholangiocarcinoma still need to be defined. Autophagy maintains cellular homeostasis by degrading defective organelles, which is an evolutionarily conserved process (Eskelinen, 2019). Autophagy can also prevent the buildup of misfolded proteins and the development of ROS. It means the inhibition of autophagy may result in autophagic cell death (Liang et al., 2020). In this study, we focused on the genetic changes in the transcription level to discover DE-ARGs between cholangiocarcinoma tissues and normal tissues. The vast majority of ARGs were up-regulated in cholangiocarcinoma, which inhibited autophagy to suppress cell proliferation and induced apoptosis in cholangiocarcinoma by triggering ROS accumulation.

Most advanced tumors exist in a circumstance with hypoxia and insufficient energy. In order to adapt to the environment, tumor tissues usually activate autophagy to maintain their high metabolic rate. Therefore, ARGs are often differentially expressed in tumor tissues and normal tissues. Fang and Chen (2020) reported that, among 487 autophagy-related genes in hepatocellular carcinoma, 60 differentially expressed genes had more than twice expression fold difference. Wang et al. (2019) reported that more than 30% of ARGs were differentially expressed in gliomas, and enrichment analysis showed that the biological process (BP) of these DE-ARGs was related to the assembly of autophagosomes. Thongchot et al. (2021) found that the expression of MAP1LC3B (LC3B, an autophagy marker gene) was up-regulated in cholangiocarcinoma and the two-year overall survival rate of patients with high expression of LC3B was higher than that of patients with low expression of LC3B from the survival curve, although there was no significant difference (*p* = 0.3761) in five-year overall survival between the two groups. As can be seen from our results, most ARGs were up-regulated in cholangiocarcinoma and the main BP was enriched in the assembly of autophagosomes. These results made it possible for us to use HCQ in the treatment of cholangiocarcinoma for the function of autophagy inhibition by HCQ that comes from the fusion suppression of autophagosomes and lysosomes (Hazari et al., 2020).

The effect of HCQ is similar to that of chloroquine, but its toxicity is only half that of chloroquine. They are used to treat tumors, initially as adjuvant drugs in combination with other antitumor drugs (Maycotte et al., 2012; Dragowska et al., 2013). It has been reported that chloroquine can enhance the antitumor effect of epirubicin. The combination of the two drugs can reduce the cell viability of lung cancer cells by about 20% and significantly increase the apoptosis of lung cancer cells (Liang et al., 2020). In addition, chloroquine can also enhance the treatment effect of some potential antitumor peptide on cervical cancer which may be related to the inhibition of autophagy. With the deepening of research, some scholars found that chloroquine or HCQ could not only be used as adjuvant drugs to treat tumors but also produce good antitumor effects as a single agent. For non-small-cell lung cancer and pancreatic cancer, the IC50 concentrations of HCQ *in vitro* were 65 μmol/L and 33 μmol/L, respectively (Nordstrøm et al., 2015). This value was 168.4 ± 23.4 μmol/L and 113.36 ± 14.06 μmol/L in HuCCT-1 and CCLP-1 cholangiocarcinoma cells according to our results, respectively, which meant that HCQ could also be extremely effective in cholangiocarcinoma treatment. When the IC50 concentration of HCQ was used for cholangiocarcinoma administration, we confirmed that the number of apoptotic cells and the expression of apoptosis-related proteins (cleaved caspase 3 and cleaved PARP1) increased significantly, accompanied by G1 phase arrest. These results implied that cell apoptosis and cycle arrest might be involved in the inhibitory effect of HCQ on cholangiocarcinoma.

The main mechanism of HCQ is to inhibit the membrane fusion of autophagosomes and lysosomes, so as to play a repression role in the final stage of autophagy (Klionsky et al., 2016; Mauthe et al., 2018). mCherry is a monomeric red fluorescent protein from mushroom coral. During the fusion of autophagosomes and lysosomes, the acidic environment within the lysosome causes the green fluorescent protein (GFP) to be quenched. When mCherry and GFP were used to label the LC3B protein together, the fluorescence in the cell will excite different colors of fluorescence depending on the autophagy state. The results from our study clearly indicated that cholangiocarcinoma cells in the control group showed scattered red spots under the background of diffuse yellow fluorescence (combined effect of mCherry and GFP) in the cytoplasm. We considered the possible reason was that when autophagosomes fused with lysosomes, the green fluorescence of GFP was partially quenched and the red fluorescence was displayed. When cholangiocarcinoma cells underwent apoptosis (IC50 concentration), the fusion of autophagosomes and lysosomes was inhibited by HCQ, while autophagy proceeded smoothly upstream. And therefore, mCherry-GFP-LC3B was aggregated on the membrane of autophagosome in the form of yellow spots under a confocal microscope. Meanwhile, the results of western blot also verified an increasing expression of autophagy-related proteins.

ROS in cells is mainly produced by electrons leaking from the mitochondrial electron transport chain (Zussman et al., 2020). It will produce a series of toxic and side effects on various cell membrane structures in cells, resulting in cell death (Hayes et al., 2020). Zhu et al. (2020) reported that administration of 20 μmol/L irinotecan for 24 h could significantly increase the ROS content in gastric cancer cells and result in apoptosis of gastric cancer cells with the help of autophagy. Other drugs (heteronemin, etc.) could also induce ROS to produce similar killing effect on cholangiocarcinoma cells (Thongsom et al., 2017; Chang et al., 2021). Similarly, HCQ induced apoptosis of cholangiocarcinoma cells with an up-regulation of ROS from our data. We suspected that necrotic and aging organelles could not be removed after autophagy was inhibited, therefore resulting in more ROS production. When GSH (a scavenger of ROS) was added, apoptosis was reduced to a certain extent, which might imply that ROS have a certain killing effect on tumor cells act as the downstream of autophagy.

Although our experiments have verified the antitumor effect of HCQ at the cellular and animal levels, our work still has some shortcomings, which are mainly reflected in animal experiments. Our results revealed that HCQ showed a good therapeutic effect on animals, but we did not further explore the detailed mechanism of HCQ *in vivo*. Considering the complexity of pharmacokinetics *in vivo* (Chen et al., 2018), we believe that the antitumor effect of HCQ *in vivo* should have its similarities and differences compared with that *in vitro*. This will be the direction we need to strive for in the future.

## Conclusion

In summary, our study indicated that HCQ induced autophagy-dependent apoptosis in cholangiocarcinoma cells *via* ROS accumulation, which could support previous evidence on the role of autophagy in the development of cholangiocarcinoma and provide some novel insights into the mechanism of the autophagy inhibitor HCQ.

## Data Availability

The original contributions presented in the study are included in the article/Supplementary Material, further inquiries can be directed to the corresponding authors.

## Abbreviations

ARGs, autophagy-related genes; CCK-8, Cell Counting Kit-8; DCF, 2′,7′-dichlorofluorescein; DE-ARGs, differential expression of autophagy-related gene sets; EdU, 5-ethynyl-2′-deoxyuridine; FCM, flow cytometry; FITC, fluorescein isothiocyanate isomer; GO, Gene Ontology; GSH, reduced l-glutathione; HCQ, hydroxychloroquine; IC50, half maximal inhibitory concentration; KEGG, Kyoto Encyclopedia of Genes and Genomes; LC3B, microtubule-associated proteins light chain 3B; PI, propidium iodine; ROS, reactive oxygen species; TCGA, The Cancer Genome Atlas; WB, western blotting.
